# Supplementary material for: Stochastic fluctuations can reveal the feedback signs of gene regulatory networks at the single-molecule level
Source: Sci Rep. 2017 Nov 22;7:16037. doi: 10.1038/s41598-017-15464-9 (PMC5700158; doi:10.1038/s41598-017-15464-9)
Supplement: Supplementary file 1 — Supplementary Information [file 41598_2017_15464_MOESM1_ESM.pdf]

Stochastic fluctuations can reveal the feedback signs of gene regulatory networks at the single-molecule level
Supplementary Information

Chen Jia[1], Peng Xie[2], Min Chen[1], Michael Q. Zhang[2,3]
[1]Department of Mathematical Sciences, University of Texas at Dallas, Richardson, TX 75080, U.S.A.
[2]Department of Biological Sciences, Center for Systems Biology, University of Texas at Dallas, Richardson, TX 75080, U.S.A.
[3]MOE Key Lab and Division of Bioinformatics, CSSB, TNLIST, Tsinghua University, Beijing 100084, China

Contents

1 Simplification of the three-stage model **1**

2 Calculation of the steady-state protein distribution **3**

3 Noise decomposition **5**

4 Upper and lower bounds for the noise in negative-feedback networks **6**

5 Upper and lower bounds for the noise in positive-feedback networks **8**

6 Estimation of the decaying rate **9**

7 Differential expression analysis **10**

1 Simplification of the three-stage model

Based on the central dogma of molecular biology, the stochastic kinetics of gene expression in a single cell can be described by the three-stage model illustrated in Fig. 1(a). The biochemical state of the gene of interest can be described by three variables (i,m,n): the activity i of its promoter, the number m of the mRNA, and the number n of the protein. Here $i=1$ and $i=0$ correspond to the active and inactive forms of the promoter, respectively. Let $p^i_{mn}(t)$ denote the probability of having m mRNAs and n proteins at time t when the promoter is in state i. Then the dynamics of the three-stage model is governed by the chemical master equation

$$\begin{cases}\dot{p}^1_{m,n} = sp^1_{m-1,n}+(m+1)vp^1_{m+1,n}+mup^1_{m,n-1}+(n+1)dp^1_{m,n+1}+a_np^0_{m,n}\\ \qquad\quad -(s+mv+mu+nd+b_n)p^1_{m,n},\\ \dot{p}^0_{m,n} = rp^0_{m-1,n}+(m+1)vp^0_{m+1,n}+mup^0_{m,n-1}+(n+1)dp^0_{m,n+1}+b_np^1_{m,n}\\ \qquad\quad -(r+mv+mu+nd+a_n)p^0_{m,n}.\end{cases}$$

Here s and r are respectively the transcription rates when the promoter is active and inactive, u is the translation rate, and v and d are respectively the degradation rates of the mRNA and protein. In

addition, a_n and b_n are the switching rates of the promoter between the active and inactive forms, which depend on the protein number n. From the viewpoint of stochastic processes, the chemical master equation is equivalent to a continuous-time Markov chain, whose transition diagram is illustrated in Fig. 1(b).

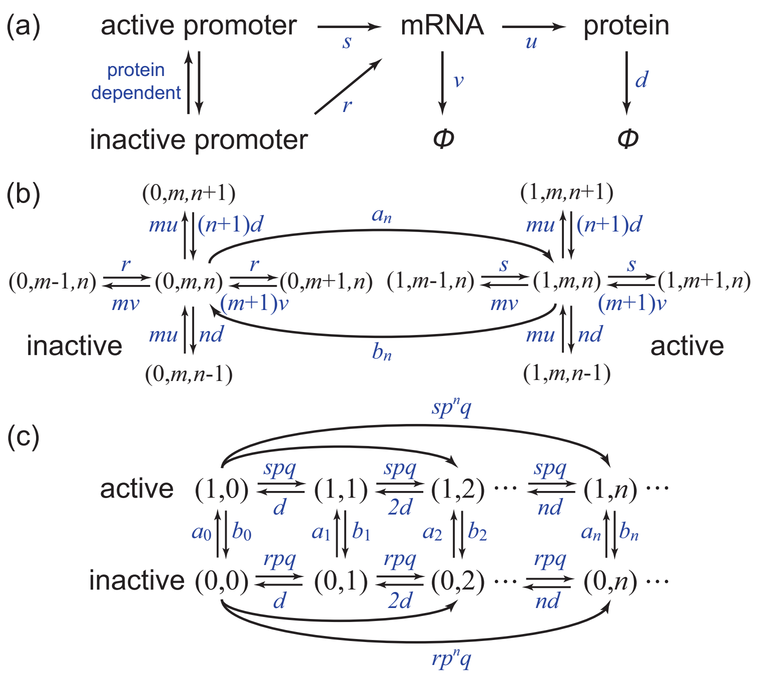

Figure 1. **Three-stage model of stochastic gene expression and its simplification.** (a) The three-stage model of stochastic gene expression in a single cell, where the promoter can switch between the active and inactive forms. (b) The transition diagram of the chemical master equation. (b) The transition diagram of the reduced model.

Experimentally, it has been consistently observed that the mRNA decays substantially faster than the protein [1, 2]. Let $\lambda = v/d$ denote the ratio of the mRNA and protein degradation rates. Under this assumption of $\lambda \gg 1$, the Markov model has two separate times scales. Let $q_{(i,m,n),(i',m',n')}$ denote the transition rate of the system from state (i, m, n) to (i', m', n') and let

$$q_{(i,m,n)} = \sum_{(i',m',n')\neq(i,m,n)} q_{(i,m,n),(i',m',n')}$$

denote the rate at which the system leaves state (i, m, n). Since $\lambda \gg 1$, we say that (i, m, n) is a fast state if $q_{(i,m,n)} \to \infty$ as $\lambda \to \infty$. Otherwise, (i, m, n) is called a slow state. If (i, m, n) is a fast state, then the leaving rate of this state will be very large and the time that the system stays in this state will be very short. Intuitively, we may expect that the original model could be simplified to a reduced model by removal of those fast states. Specifically, it is easy to check that

$$q_{(0,m,n)} = mu + md\lambda + s + nd + a_n, \quad q_{(1,m,n)} = mu + md\lambda + s + nd + b_n.$$

This shows that all the states (i, m, n) with $m \geq 1$ are fast states and can be removed. Equivalently, all the states $(i, 0, n)$ are slow states and are retained.

Let A denote the set of all the slow states and let B denote the set of all the fast states. By relabelling the states, the transition rate matrix Q of the original model can be represented as the block matrix

$$Q = \begin{pmatrix} Q_{AA} & Q_{AB} \\ Q_{BA} & Q_{BB} \end{pmatrix}.$$

According to a recently developed simplification method of two-time-scale Markov chains [3, 4], the original model can be simplified to an reduced model by removal of the fast states. The state space of the reduced model is the slow state space A and the transition rate matrix $\tilde{Q}$ of the reduced model is given by

$$\tilde{Q} = Q_{AA} - Q_{AB}Q_{BB}^{-1}Q_{BA}.$$

By using this formula, the effective transition rates of the reduced model can be calculated, as illustrated in Fig. 1(c). In the reduced model, the effect of transcription is coarse-grained and the biochemical state of the gene is only described by the variables i and n. Let $p_{i,n}(t)$ denote the probability of having n proteins at time t when the promoter is in state i. Then the evolution of the reduced model is governed by the chemical master equation

$$\begin{cases} \dot{p}_{1,n} = \sum_{k=1}^{n-1} sp^{n-k}qp_{1,k} + (n+1)dp_{1,n+1} + a_n p_{0,n} - (sp + nd + b_n)p_{1,n}, \\ \dot{p}_{0,n} = \sum_{k=1}^{n-1} rp^{n-k}qp_{0,k} + (n+1)dp_{0,n+1} + b_n p_{1,n} - (rp + nd + a_n)p_{0,n}, \end{cases}$$

where $p = u/(u+v)$ and $q = v/(u+v)$.

It should be pointed out that the reduced model includes long-range interactions of protein numbers, which means that protein synthesis occurs in random bursts. The rate at which k proteins are synthesized by an mRNA is sp^kq when the promoter is active and is rp^kq when the promoter is inactive. This fact can be understood intuitively. When the promoter is active, the transcription rate is s. Once an mRNA is synthesized, it can either synthesize protein with probability $p = u/(u+v)$ or degrade with probability $q = v/(u+v)$. Since the mRNA dynamics is fast, the probability that the mRNA can produce k proteins before it finally degrades will be p^kq, which follows the geometric distribution. Overall, the effective rate at which k proteins are synthesized when the promoter is active will be the product of the transcription rate s and the geometric probability p^kq.

2 Calculation of the steady-state protein distribution

In most applications, the switching rates of the promoter are fast [1]. Since $a_n, b_n \gg 1$, the two states $(0,n)$ and $(1,n)$ of the reduced model can be aggregated into a single state. In this way, the reduced model can be further simplified to the Markov model illustrated in Fig. 2, in which the biochemical state of the gene is only described by the protein number n.

The remaining question is to calculate the effective transition rates of the simplified model. Since $a_n, b_n \gg 1$, the two states $(0,n)$ and $(1,n)$ of the reduced model will reach a quasi-steady

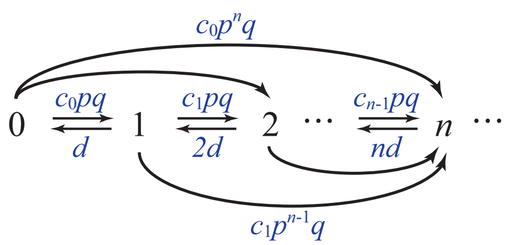

Figure 2. Simplified model when $a_n, b_n \gg 1$.

state with quasi-steady-state distribution

$$p^{qss}_{(1,n)} = \frac{a_n}{a_n + b_n}, \quad p^{qss}_{(0,n)} = \frac{b_n}{a_n + b_n}.$$

For convenience, set

$$c_n = \frac{a_n s + b_n r}{a_n + b_n},$$

Then the effective transition rate from state n to $n + k$ is given by

$$\hat{q}_{n,n+k} = p^{qss}_{(1,n)}\tilde{q}_{(1,n),(1,n+k)} + p^{qss}_{(0,n)}\tilde{q}_{(0,n),(0,n+k)} = c_n p^k q$$

and the effective transition rate from state n to $n - 1$ is given by

$$\hat{q}_{n,n-1} = p^{qss}_{(1,n)}\tilde{q}_{(1,n),(1,n-1)} + p^{qss}_{(0,n)}\tilde{q}_{(0,n),(0,n-1)} = nd.$$

Let $p_n(t)$ denote the probability of having n proteins at time t and let p^{ss}_n denote the corresponding steady-state probability. Then the evolution of the simplified model is governed by the chemical master equation

$$\dot{p}_n = \sum_{k=1}^{n-1} c_k p^{n-k} q p_k + (n+1)dp_n + 1 - (c_n p + nd)p_n. \tag{1}$$

Recall that the steady-state probability p^{ss}_n must satisfy

$$(\hat{q}_{n,n-1} + \sum_{k=1}^{\infty} \hat{q}_{n,n+k})p^{ss}_n = \sum_{k=0}^{n-1} \hat{q}_{k,n} p^{ss}_k, \tag{2}$$

where $\hat{q}_{kl}$ is the transition rate from state k to l. By solving Eq. (4), the steady-state distribution of the protein copy number is given by

$$p^{ss}_n = A\frac{p^n}{n!}\frac{c_0}{d}\left(\frac{c_1}{d} + 1\right)\cdots\left(\frac{c_{n-1}}{d} + n - 1\right), \tag{3}$$

where A is a normalization constant.

3 Noise decomposition

From Eq. (1), it is easy to check that the mean $\langle n \rangle$ and variance σ^2 of the protein copy number satisfy the following set of ordinary differential equations

$$\begin{cases} \dfrac{\mathbf{d}\langle n \rangle}{\mathbf{d}t} = -d\langle n \rangle + \dfrac{p}{q}\langle c_n \rangle, \\ \dfrac{\mathbf{d}\sigma^2}{\mathbf{d}t} = -2d\sigma^2 + \dfrac{2p}{q}\text{Cov}(n, c_n) + d\langle n \rangle + \left(\dfrac{2p}{q^2} - \dfrac{p}{q}\right)\langle c_n \rangle, \end{cases}$$

where $\text{Cov}(n, c_n) = \langle nc_n \rangle - \langle n \rangle\langle c_n \rangle$ is the covariance between n and c_n. We stress here that given Eq. (1), the above two moments equations hold accurately without any approximation, even when the nonlinearity of feedback regulation is very high. At the steady state, it is easy to check that

$$\langle n \rangle = \frac{p}{dq}\langle c_n \rangle, \quad \sigma^2 = \frac{1}{q}\langle n \rangle + \frac{p}{dq}\text{Cov}(n, c_n). \tag{4}$$

From these two equations, the steady-state noise η in the protein number is given by

$$\eta = \frac{\sigma^2}{\langle n \rangle^2} = \frac{1}{q\langle n \rangle} + \eta_f, \tag{5}$$

where

$$\eta_f = \frac{\text{Cov}(n, c_n)}{\langle n \rangle\langle c_n \rangle} = \frac{1}{\langle n \rangle\langle c_n \rangle}\sum_n (n - \langle n \rangle)c_n p_n.$$

The noise decomposition formula (5) can be rewritten into a more illuminating form. On one hand, the average number of proteins synthesized per unit time is $\langle m \rangle u$. On the other hand, the average number of proteins degraded per unit time is $\langle n \rangle d$. At the steady state, these two quantities should cancel out, which means that $\langle m \rangle u = \langle n \rangle d$. This indicates that $d/v\langle m \rangle = u/v\langle n \rangle = p/q\langle n \rangle$. Therefore, Eq. (5) can be rewritten as

$$\eta = \frac{1}{\langle n \rangle} + \frac{d}{v\langle m \rangle} + \eta_f.$$

If the network has no feedback, c_n is a constant and thus $\eta_f = 0$. If the network has a positive-feedback loop, c_n is an increasing function of n. This shows that $c_n \geq c_{\langle n \rangle}$ when $n \geq \langle n \rangle$, while $c_n < c_{\langle n \rangle}$ when $n < \langle n \rangle$. This further suggests that

$$\begin{aligned} \text{Cov}(n, c_n) &= \sum_n (n - \langle n \rangle)c_n p_n = \sum_{n \geq \langle n \rangle} (n - \langle n \rangle)c_n p_n + \sum_{n < \langle n \rangle} (n - \langle n \rangle)c_n p_n, \\ &> c_{\langle n \rangle} \sum_{n \geq \langle n \rangle} (n - \langle n \rangle)p_n + c_{\langle n \rangle} \sum_{n < \langle n \rangle} (n - \langle n \rangle)p_n = 0, \end{aligned}$$

and thus $\eta_f > 0$. Similarly, if the network has a negative-feedback loop, c_n is a decreasing function of n and thus $\eta_f < 0$.

4 Upper and lower bounds for the noise in negative-feedback networks

In this section, we shall provide the bounds for the noise in the negative-feedback case. By the Cauchy-Schwarz inequality, we obtain that

$$\eta_f = \frac{\mathrm{Cov}(n, c_n)}{\langle n \rangle \langle c_n \rangle} \geq -\frac{\sqrt{\mathrm{Var}(n)}}{\langle n \rangle} \frac{\sqrt{\mathrm{Var}(c_n)}}{\langle c_n \rangle} = -\sqrt{\eta}\sqrt{\eta_{c_n}},$$

where $\eta_{c_n} = \mathrm{Var}(c_n)/\langle c_n \rangle^2$ is the steady-state noise of the effective transcription rate c_n. This inequality, together with Eq. (5), shows that

$$\eta \geq \frac{1}{q\langle n \rangle} - \sqrt{\eta}\sqrt{\eta_{c_n}}, \tag{6}$$

which gives a lower bound for the protein noise. It is written in a different form but is essentially equivalent to the lower bound obtained in [5]. It is indispensable to notice that this lower bound includes the information of η itself and may be even negative in the regime of strong noise suppression. In the following, we shall give a better lower bound for the noise η which is always positive and only requires the knowledge on original model parameters.

To this end, we introduction some notations. Let $c(x)$ be the function obtained from c_n by replacing n with a positive real number x. Let $c'(x)$ be the derivative of $c(x)$ and let

$$\alpha = \sup_{x \geq 0} |c'(x)|$$

be the supremum norm of the function $c'(x)$. Let X and Y be two independent random variables such that $P(X = n) = P(Y = n) = p_n^{ss}$ for all $n \geq 0$. In other words, X and Y are independent and have the same distribution as the steady-state protein number n. According to Lagrange's mean value theorem, we have

$$(c(X) - c(Y))^2 = c'(\xi)^2(X - Y)^2 \leq \alpha^2(X - Y)^2.$$

where ξ is some value between X and Y. Taking expectation on both sides of this equality and noting that X and Y are independent and identically distributed, gives rise to

$$\langle c(X)^2 \rangle - \langle c(X) \rangle^2 \leq \alpha^2[\langle X^2 \rangle - \langle X \rangle^2].$$

Since X has the same distribution as the steady-state protein number n, we have

$$\mathrm{Var}(c_n) \leq \alpha^2 \mathrm{Var}(n).$$

From Eq. (4), it is easy to see that

$$\langle c_n \rangle = \frac{dq}{p}\langle n \rangle.$$

This indicates that

$$\eta_{c_n} = \frac{\mathrm{Var}(c_n)}{\langle c_n \rangle^2} \leq \left(\frac{\alpha p}{dq}\right)^2 \frac{\mathrm{Var}(n)}{\langle n \rangle^2} = \left(\frac{\alpha p}{dq}\right)^2 \eta.$$

Inserting this inequality into Eq. (6) gives rise to

$$\eta \geq \frac{1}{q\langle n\rangle} - \frac{\alpha p}{dq}\eta.$$

Therefore, the noise η has a lower bound which is given by

$$\eta \geq \frac{1}{q\langle n\rangle}\frac{1}{1+\alpha p/dq}.$$

Since $\eta_f < 0$, it follows from Eq. (5) that

$$\frac{1}{q\langle n\rangle}\frac{1}{1+\alpha p/dq} \leq \eta < \frac{1}{q\langle n\rangle}. \tag{7}$$

In the literature, the promoter switching rates are often chosen as $a_n = \mu$ and $b_n = \nu n^h$ with $h \geq 1$. In this case, the regulatory function $c(x)$ is the generalized Hill function

$$c(x) = \frac{sa + rx^h}{a + x^h},$$

where $a = \mu/\nu$ and h is the Hill coefficient. It is easy to check that

$$c'(x) = -(s-r)ah\frac{x^{h-1}}{(a+x^h)^2}.$$

Recall that the maximum point x_0 of the function $c'(x)$ must satisfy $c''(x_0) = 0$. Direct calculation shows that

$$c''(x) = (s-r)ahx^{h-2}\frac{(h+1)x^h - (h-1)a}{(a+x^h)^3}.$$

By solving $c''(x_0) = 0$, we obtain that

$$x_0^h = \frac{h-1}{h+1}a.$$

This shows that

$$\alpha = -c'(x_0) = (s-r)ah\frac{x_0^{h-1}}{(a+x_0^h)^2} = \frac{(h-1)^{1-1/h}(h+1)^{1+1/h}}{4h}\frac{s-r}{a^{1/h}}.$$

In Eq. (7), the term $\alpha p/dq$ is of crucial importance. If $c(x)$ is the generalized Hill function, then

$$\frac{\alpha p}{dq} = \frac{(h-1)^{1-1/h}(h+1)^{1+1/h}}{4h}a^{-1/h}\frac{(s-r)}{d}\frac{p}{q}.$$

Here the term $(s-r)/d$ can be understood as the typical number of mRNAs in a single cell. Recall that the probability that an mRNA can produce k proteins before it finally degrades follows the geometric distribution p^kq. Therefore, the mean burst size of the protein is

$$\sum_{k=0}^{\infty} kp^kq = \frac{p}{q}.$$

Therefore, the term $(s-r)p/dq$ is the typical number of proteins in a single cell. Recall that the promoter switching rates are given by $a_n = \mu$ and $b_n = \nu n^h$. In most applications, these two quantities should have the same order of magnitude, that is,

$$\mu \sim \nu \left(\frac{(s-r)p}{dq}\right)^h.$$

Since $a = \mu/\nu$, we have

$$a^{1/h} \sim \frac{(s-r)p}{dq}.$$

This indicates that the term $\alpha p/dq$ is of the order of 1 for a wide range of biologically relevant parameters.

5 Upper and lower bounds for the noise in positive-feedback networks

In this section, we shall provide the bounds for the noise in the positive-feedback case. By the Cauchy-Schwarz inequality, it is easy to check that

$$\eta \leq \frac{1}{q\langle n\rangle} + \sqrt{\eta}\sqrt{\eta_{c_n}}. \tag{8}$$

Similarly, we can also prove that

$$\eta_{c_n} \leq \left(\frac{\alpha p}{dq}\right)^2 \eta.$$

Inserting this inequality into Eq. (8) gives rise to

$$\eta \leq \frac{1}{q\langle n\rangle} + \frac{\alpha p}{dq}\eta.$$

If $\alpha p < dq$, then the noise η has an upper bound which is given by

$$\eta \leq \frac{1}{q\langle n\rangle}\frac{1}{1-\alpha p/dq}.$$

Since $\eta_f > 0$, it follows from Eq. (5) that

$$\frac{1}{q\langle n\rangle} < \eta \leq \frac{1}{q\langle n\rangle}\frac{1}{1-\alpha p/dq}.$$

In the literature, the promoter switching rates are often chosen as $a_n = \mu n^h$ with $h \geq 1$ and $b_n = \nu$. In this case, the regulatory function $c(x)$ is the generalized Hill function

$$c(x) = \frac{ra + sx^h}{a + x^h}.$$

where $a = \nu/\mu$. Let x_0 be the maximum point of the function $c'(x)$. By solving $c''(x_0) = 0$, we obtain that

$$x_0^h = \frac{h-1}{h+1}a.$$

This shows that

$$\alpha = c'(x_0) = (s-r)ah\frac{x_0^{h-1}}{(a+x_0^h)^2} = \frac{(h-1)^{1-1/h}(h+1)^{1+1/h}}{4h}\frac{s-r}{a^{1/h}}.$$

6 Estimation of the decaying rate

Assume that $n \gg 1$ is a fixed protein copy number. In the main text, we have shown that

$$p_{n+k}^{ss} \approx p^k p_n^{ss} = e^{k \log p} p_n^{ss}. \tag{9}$$

Since the mean burst size p/q of the protein is relatively large in living cells, we have $q \ll p$. Therefore, Eq. (9) shows that $-\log p = -\log(1-q) \approx q$ is the decaying rate for the steady-state distribution of the protein copy number.

In single-cell experiments such as flow cytometry, data of protein concentrations, instead of protein copy numbers, are usually measured. Let $x = n/V$ be a continuous variable representing the concentration of the protein, where V is a constant compatible with the macroscopic scale. In flow cytometry, $1/V$ stands for the fluorescence per protein molecule. Since x is a continuous variable, we need to choose an arbitrary step size h. If we plot the histogram of the steady-state data of protein concentrations with step size h, then $u_n = P(x \in [nh, (n+1)h])$ is the height of the nth bin. When $n \gg 1$, it follows from Eq. (9) that

$$u_n = \sum_{m \in [nhV, (n+1)hV]} p_m^{ss} \approx hV p_{nhV} \sum_{k=0}^{hV} p^m.$$

Similarly, we have

$$u_{n+k} \approx hV p_{(n+k)hV} \sum_{k=0}^{hV} p^m.$$

Therefore, it follows from Eq. (9) that

$$u_{n+k} = u_n p^{khV} = u_n e^{khV \log p}.$$

This suggests that $-V \log p \approx qV$ is the decaying rate for the steady-state distribution of the protein concentration. Taking logarithm on both sides of this equation gives rise to

$$-\log u_{n+k} \approx -\log u_n + khqV.$$

This is a linear equation with respect to k and can be used to estimate the decaying rate qV. We only need to draw the histogram of the protein concentration, calculate the height u_n of each bin, and then perform a linear regression analysis between n and $-\log u_n$ when $n \gg 1$. The slope of the linear regression analysis is exactly hqV, from which we can obtain an robust estimation of the decaying rate qV.

We performed the above data analysis on steady-state single-cell fluorescence data of the zsGreen and rsRed proteins measured by flow cytometry. To filter out data from dead cells, we excluded samples with extremely low fluorescence. We divided the fluorescence data into many bins with the same step size h and appropriately chose an interval $[x, 2x]$ with x large enough such that the logarithmic height $-\log u_n$ of each bin in this interval arranges as an approximate linear function of n. For the zsGreen protein, the step size h is chosen between 24 a.u. to 120 a.u. For the

rsRed protein, the step size is chosen between 5 a.u. to 12 a.u. In the high Dox case, the logarithmic heights $-\log u_n$ of the zsGreen protein versus the sequence numbers of bins under different IPTG concentrations are depicted in Fig. 3. We calculated the slope hqV using Matlab and then estimated the decaying rate qV. Similarly, we can also estimate the the decaying rate for the zsGreen and rsRed proteins in the high and low Dox cases.

7 Differential expression analysis

Since feedback regulation significantly affects noise, it may give rise to bias in the noise-based differential expression analysis. In recent years, the negative-binomial (NB) model has been widely used as the null model to identify differentially expressed genes (DEGs). The NB model is a special case of our model when the network has no feedback. The effect of noise amplification or reduction caused by feedback regulation is not addressed under the NB assumption, which may result in incorrect predictions.

To see this weakness of the NB model, we performed pairwise differential expression analysis across the IPTG concentration. We merged fluorescence data of the zsGreen protein under two different IPTG concentrations to form the sample of a *bona-fide* DEG. In the NB model, c_n is a constant and the feedback coefficient η_f vanishes. Therefore, the protein noise estimated by the NB model is given by

$$\hat{\eta} = \frac{1}{q\langle n\rangle}.$$

The observed noise η and the null-model noise $\hat{\eta}$ of many DEGs were plotted in Fig. 4e of the main text and Fig. 4. Since IPTG has significant effect on the synthetic gene circuit, each DEG should result in larger observed noise than the null-model noise. The data points under the diagonal of Fig. 4e imply the weakness of the NB model in dealing with genes with feedback regulation.

References

[1] Friedman N, Cai L, Xie XS. Linking stochastic dynamics to population distribution: an analytical framework of gene expression. Phys Rev Lett. 2006;97(16):168302.

[2] Shahrezaei V, Swain PS. Analytical distributions for stochastic gene expression. Proc Natl Acad Sci USA. 2008;105(45):17256–17261.

[3] Jia C. Reduction of Markov chains with two-time-scale state transitions. Stochastics. 2016;88(1):73–105.

[4] Jia C. Simplification of irreversible Markov chains by removal of states with fast leaving rates. J Theor Biol. 2016;400:129–137.

[5] Hilfinger A, Norman TM, Vinnicombe G, Paulsson J. Constraints on fluctuations in sparsely characterized biological systems. Physical review letters. 2016;116(5):058101.

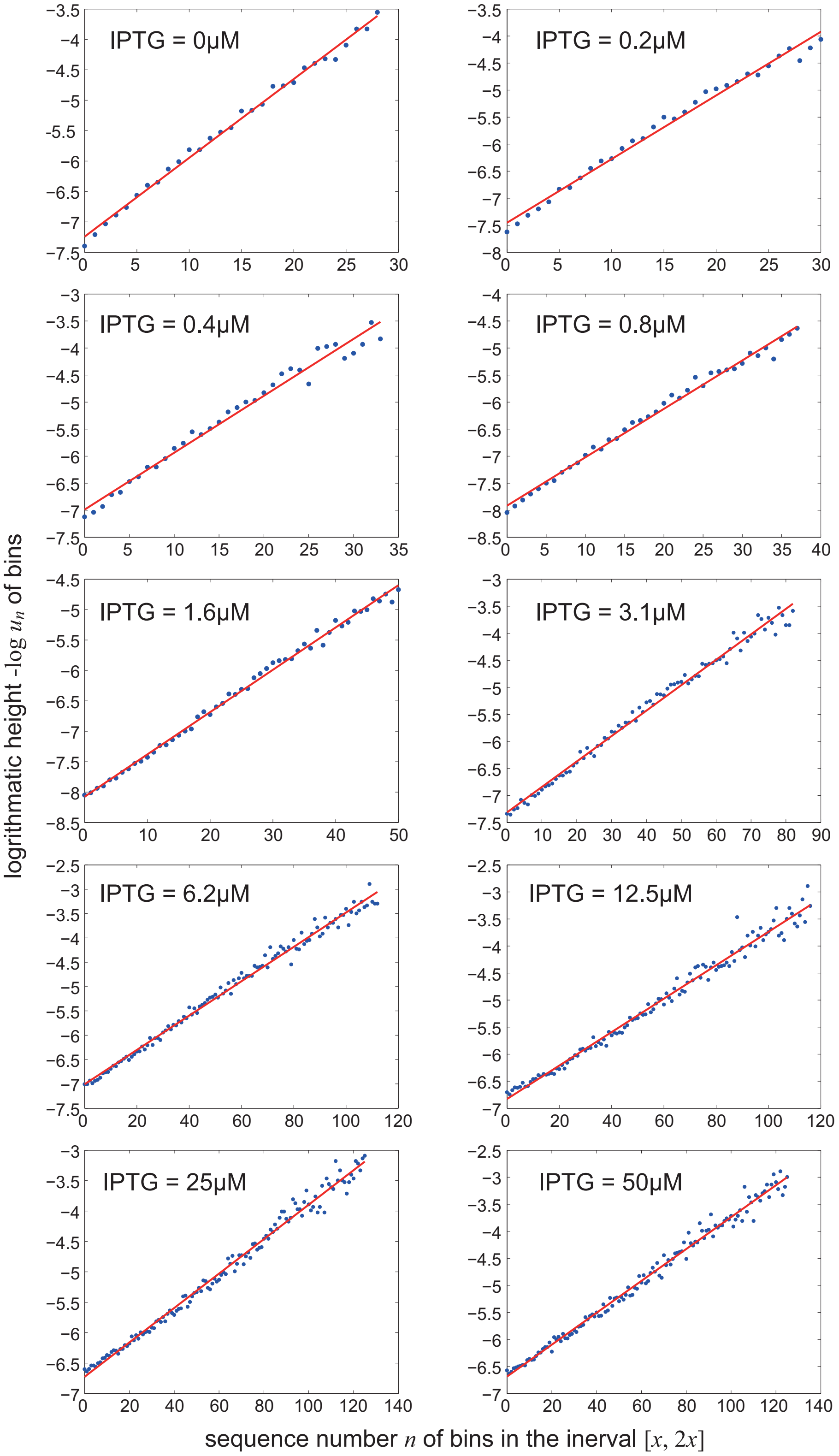

Figure 3. Estimation of the decaying rate for the steady-state distribution of the zsGreen fluorescence under different IPTG concentration in the high Dox case. The x-axis represents the sequence number n of bins in the interval $[x, 2x]$ and the y-axis represents the logarithmic height $-\log u_n$ of these bins.

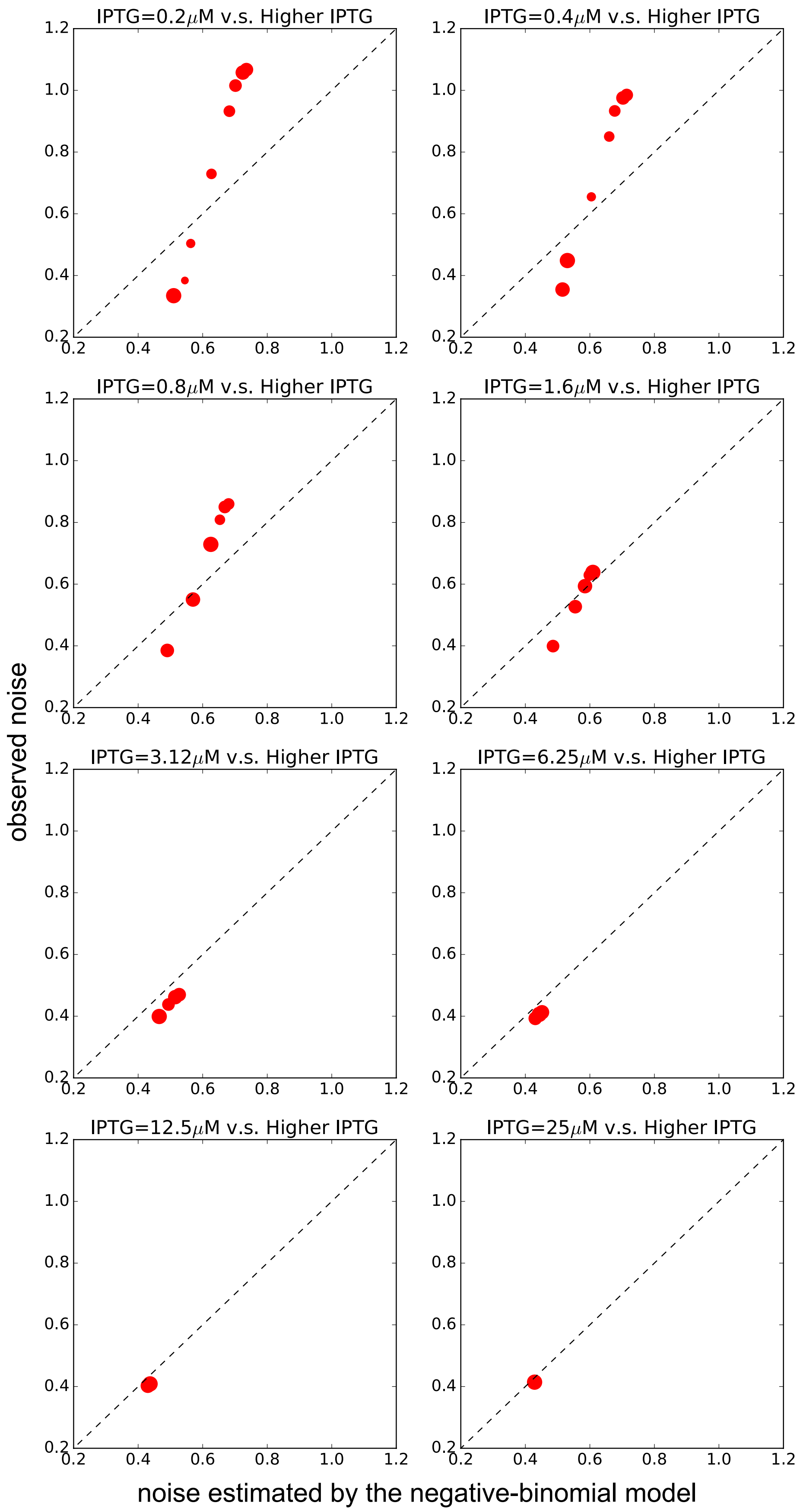

Figure 4. The observed noise versus the noise estimated by the NB model for different DEGs in the negative-feedback case. Each red circle represents a bona-fide DEG whose expression data are generated by merging the data of the zsGreen protein under two different IPTG concentrations. The size of the red circle is proportional to the difference of the two IPTG concentrations.
